# Desquamative Gingivitis and the Oral Microbiome: Insights into Immune–Microbial Interactions in Mucosal Inflammation

**DOI:** 10.3390/dj13110541

**Published:** 2025-11-17

**Authors:** Bruno Špiljak, Ana Andabak Rogulj, Božana Lončar Brzak, Vlaho Brailo, Ivana Škrinjar, Petar Ozretić, Danica Vidović Juras

**Affiliations:** 1 Department of Oral Medicine, University of Zagreb School of Dental Medicine, 10000 Zagreb, Croatia; bspiljak@sfzg.unizg.hr (B.Š.); andabak@sfzg.unizg.hr (A.A.R.); loncar@sfzg.unizg.hr (B.L.B.); brailo@sfzg.unizg.hr (V.B.); iskrinjar@sfzg.unizg.hr (I.Š.); 2 Clinical Department of Oral Diseases, Dental Clinic, University Hospital Centre (UHC), 10000 Zagreb, Croatia; 3 Laboratory for Hereditary Cancer, Division of Molecular Medicine, Ruđer Bošković Institute, 10000 Zagreb, Croatia; petar.ozretic@irb.hr

**Keywords:** desquamative gingivitis, oral lichen planus, oral microbiome, dysbiosis, mucous membrane pemphigoid, pemphigus vulgaris, mucosal immunity, immune–microbial interactions, oral mucosal diseases

## Abstract

Desquamative gingivitis (DG) is a clinical presentation characterized by erythema, epithelial desquamation, and mucosal fragility, commonly associated with immune-mediated diseases such as oral lichen planus (OLP), mucous membrane pemphigoid (MMP), and pemphigus vulgaris (PV). While traditionally viewed as a manifestation of immune dysregulation, growing evidence suggests that the oral microbiome may modulate disease onset, persistence, and severity. This review summarizes current knowledge on the oral microbiota in DG and its underlying diseases, explores mechanistic links between dysbiosis and immune activation, and discusses clinical and research implications. A narrative literature review was conducted using PubMed and Scopus, focusing on studies analyzing the oral microbiome in OLP, MMP, and PV. Emphasis was placed on molecular microbiology techniques, immune profiling, and functional or longitudinal approaches. In OLP, microbial dysbiosis is consistently reported, including reduced diversity and increased abundance of pro-inflammatory genera such as *Fusobacterium*, *Prevotella*, and *Capnocytophaga*. These shifts correlate with epithelial barrier disruption and inflammatory cytokine production. Although data on MMP and PV are limited, early findings suggest microbial involvement in sustaining inflammation, delaying healing, and possibly amplifying autoimmune responses. Dysbiosis may activate Toll-like receptors, skew T cell responses, and contribute to the breakdown of immune tolerance. DG may reflect a dynamic interplay between immune mechanisms and microbial ecology. While evidence is strongest for OLP, preliminary data suggest broader microbial contributions across DG-associated diseases. Microbiome-informed approaches could enhance diagnostic accuracy and support the development of adjunctive therapies.

## 1. Introduction

Desquamative gingivitis (DG) is a clinical descriptor, not a definitive diagnosis, characterized by erythematous, friable, and often painful gingiva that bleeds easily upon minimal mechanical stimulation [1,2,3]. The term emphasizes its descriptive nature, since clinicians are more often confronted with this nonspecific mucosal appearance rather than a standalone disease. Rather than a distinct disease entity, DG reflects a common mucosal manifestation of several systemic and autoimmune disorders, most notably oral lichen planus (OLP), mucous membrane pemphigoid (MMP), and pemphigus vulgaris (PV). These three conditions together account for the vast majority of cases encountered in practice, underscoring the need for careful differential diagnosis. Less frequently, DG may also be associated with other bullous disorders, chronic graft-versus-host disease, and lupus erythematosus, conditions that can present with overlapping clinical features and therefore add to diagnostic complexity [1,2,3,4,5].

The pathogenesis of these underlying conditions is primarily immunologically mediated, featuring T-cell dysregulation, autoantibody formation, and epithelial–connective tissue interface damage [2,3,6,7,8]. These mechanisms result in chronic inflammation, impaired barrier function, and progressive mucosal fragility. Importantly, the gingival tissues are constantly exposed to mechanical stimuli, microbial colonization, and immune surveillance, which together create a unique microenvironment where autoimmune mechanisms can be amplified. However, recent studies have suggested that the oral microbiota may act as a modulator or amplifier of local inflammation, particularly in the context of immune-mediated oral diseases [2,9,10,11,12,13]. This implies that microbial communities might not only serve as secondary triggers but also play a role in sustaining chronic inflammation through continuous crosstalk with immune cells.

The concept that microbial communities influence mucosal immune responses, epithelial barrier integrity, and disease persistence is well established in gastrointestinal disorders such as inflammatory bowel disease, but is now gaining traction in oral mucosal diseases, including DG [10,12,14,15,16]. In both gastrointestinal and oral contexts, disruption of microbial homeostasis can lead to aberrant immune activation, loss of tolerance, and tissue destruction. Thus, DG may represent a localized manifestation of a broader phenomenon in which mucosal surfaces and their resident microbiota are intimately linked in health and disease.

As high-throughput sequencing technologies evolve, a growing number of studies have begun to explore the composition and function of the gingival microbiome in health and disease [17,18,19,20,21,22,23]. These advances have revealed complex microbial networks, dynamic shifts between symbiosis and dysbiosis, and functional consequences of microbial alterations at mucosal surfaces. Notably, oral lichen planus has been associated with dysbiosis, characterized by reduced microbial diversity and increased abundance of genera such as *Fusobacterium* and *Prevotella* in erosive forms [24,25,26,27,28,29]. Such findings provide a microbial perspective that complements the well-described immunological abnormalities in OLP and suggest that the microbiome could influence clinical phenotypes and disease severity. However, the specific role of the oral microbiota in immune-mediated desquamative conditions remains underexplored, particularly in disorders beyond OLP such as MMP and PV, where microbiome data are almost absent [30]. The paucity of data in these entities highlights the need for integrative approaches that combine immunological profiling with microbial characterization.

The aim of this review is therefore to summarize the current evidence on the oral microbiome in desquamative gingivitis, focusing on its possible involvement in disease onset, progression, and persistence. In addition, we explore how microbial–immune interactions may contribute to tissue damage and consider potential diagnostic and therapeutic implications for future clinical care. By bridging microbiological and immunological perspectives, this review seeks to provide a more comprehensive understanding of DG and highlight avenues for precision-based interventions.

## 2. Desquamative Gingivitis: Clinical and Pathological Features

DG is best understood as a clinical manifestation rather than a stand-alone diagnosis, most frequently encountered in patients with chronic mucocutaneous disorders. Epidemiological data indicate that DG predominantly affects women over the age of 40 and most commonly involves the facial gingiva of the anterior teeth, although virtually any gingival site can be affected [1,2,3,5,7,31]. This demographic distribution suggests potential contributions of hormonal influences, immune dysregulation, and genetic susceptibility, while the predilection for anterior regions may also reflect greater exposure to mechanical trauma, accumulation of dental biofilm, and chronic irritation in areas of high functional demand.

Clinically, DG presents with erythema, epithelial desquamation, erosions, and ulcerations. Patients frequently report pain, burning sensations, and gingival bleeding, particularly during toothbrushing or mastication, which can substantially impair oral function and quality of life. The gingiva often appears shiny, atrophic, and friable, with epithelial sloughing that exposes inflamed connective tissue beneath. The severity and distribution of lesions are highly variable and largely dependent on the underlying disease and the degree of immune dysregulation [1,2,3,4,5]. Repeated microtrauma and routine oral hygiene measures can exacerbate symptoms (Koebner-like phenomenon), while secondary infection and poor plaque control may further complicate the clinical picture by amplifying inflammatory changes.

The three major immune-mediated conditions underlying DG illustrate distinct but overlapping pathogenic mechanisms. In OLP, a T cell-mediated chronic inflammatory disorder of uncertain etiology, histopathology typically reveals hyperkeratosis, basal cell degeneration, and a dense band-like lymphocytic infiltrate in the lamina propria. DIF frequently demonstrates fibrinogen deposition along the basement membrane zone, reinforcing the role of cell-mediated immunity in disrupting epithelial integrity [32,33,34]. In MMP, a chronic autoimmune subepithelial blistering disorder, autoantibodies target structural proteins of the basement membrane. Histology demonstrates subepithelial clefting, while DIF reveals linear deposits of IgG, IgA, and/or C3 along the basement membrane, reflecting antibody-mediated compromise of epithelial–connective tissue adhesion [35,36,37]. In PV, circulating autoantibodies against desmoglein-3 interfere with desmosomal adhesion, leading to intraepithelial blistering. Histopathology shows suprabasal acantholysis with detached keratinocytes, whereas DIF demonstrates the classic intercellular “fishnet” pattern of IgG deposition throughout the epithelium [37,38]. While the clinical appearance of DG in these disorders may be remarkably similar, the underlying histopathological and immunopathological signatures highlight their distinct immunological targets—desmosomes in PV, basement membrane components in MMP, and T cell-mediated basal cell injury in OLP.

**Figure 1:**
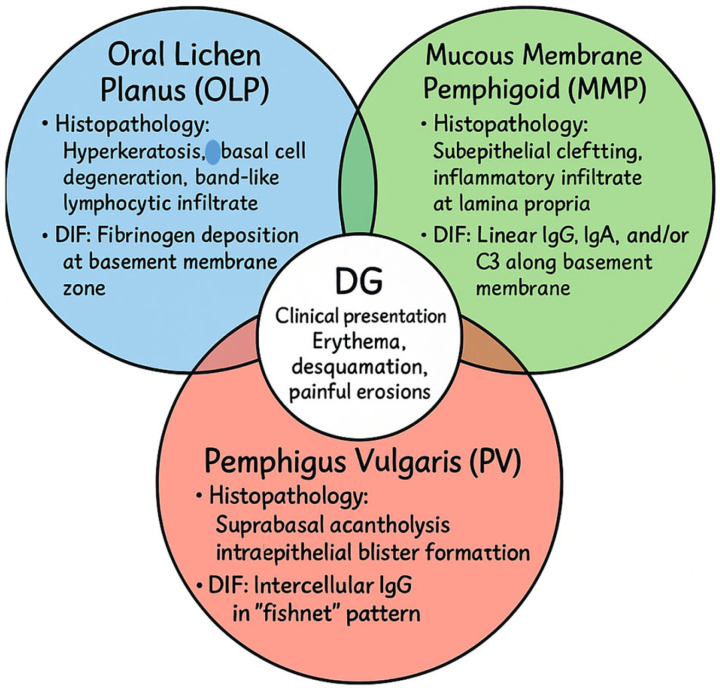
Clinical, histopathological, and immunofluorescence features of the most common diseases presenting with desquamative gingivitis, based on the current literature data [32,33,34,35,36,37,38].

Because of this significant clinical overlap, accurate diagnosis requires an integrated approach combining clinical assessment, histopathological examination, and immunofluorescence, while excluding mimicking conditions such as lupus erythematosus, chronic graft-versus-host disease, and other autoimmune blistering disorders [3,5] (Figure 1). Such a stepwise strategy is essential to minimize misclassification and to enable the initiation of tailored therapies at earlier stages of disease.

A multidisciplinary framework that incorporates oral medicine, dermatology, and pathology is therefore indispensable in identifying the underlying disorder and guiding patient management. Beyond conventional diagnostic approaches, recent evidence suggests that the oral microbiome may significantly modulate mucosal immune responses, influencing both disease activity and chronicity. Consequently, investigation of the microbial environment in DG may not only enhance diagnostic accuracy but also inform therapeutic strategies, particularly as precision medicine begins to integrate host–microbiome interactions into treatment planning [10,12,21].

## 3. Oral Microbiome in Gingival Health and Disease

The human oral cavity harbors one of the most diverse and complex microbial ecosystems in the body, with more than 700 bacterial species identified across distinct ecological niches, including the tongue, buccal mucosa, hard palate, and gingiva [12,39,40,41]. This remarkable diversity reflects the oral cavity’s unique structural and functional environment, which combines hard surfaces such as teeth with constantly shedding mucosal epithelia, providing multiple microhabitats for microbial colonization. In health, the gingival microbiome is dominated by commensal genera such as *Streptococcus*, *Actinomyces*, *Veillonella*, *Granulicatella*, and *Rothia*. These taxa contribute not only to nutrient cycling but also to the maintenance of epithelial barrier homeostasis, the induction of immune tolerance, and colonization resistance against invading pathogens [12,21,42,43,44]. Through continuous crosstalk with the host, commensal organisms stimulate regulatory immune pathways, produce metabolites that reinforce barrier integrity, and outcompete potential pathogens, thereby sustaining a state of symbiosis.

When gingival inflammation develops, as seen in gingivitis and periodontitis, the microbial community undergoes a profound shift toward dysbiosis. This transition is characterized by an increased abundance of proteolytic, anaerobic, and pro-inflammatory taxa, including *Fusobacterium nucleatum*, *Porphyromonas gingivalis*, *Prevotella intermedia*, and *Treponema denticola* [43,45,46,47]. Such organisms are capable of evading immune clearance, producing virulence factors that degrade extracellular matrices, and disrupting epithelial junctions. The resulting ecological imbalance creates a self-perpetuating cycle: inflammation alters the local microenvironment by providing heme, peptides, and other nutrients that favor the growth of pathogenic bacteria, while dysbiosis further amplifies tissue-damaging inflammatory responses [48,49,50,51]. This bidirectional relationship underscores the dynamic nature of host–microbe interactions in gingival tissues, where health and disease are separated by fragile ecological boundaries.

While the role of dysbiosis in periodontitis is firmly established, its contribution to immune-mediated gingival diseases such as DG remains less clearly defined. Insights can be gained from systemic inflammatory conditions, including inflammatory bowel disease and psoriasis, where microbial communities are increasingly recognized as active participants in shaping disease susceptibility, persistence, and severity [52,53,54]. These parallels suggest that the gingival microbiome may similarly modulate host immune pathways in conditions where autoimmunity or chronic inflammation drives epithelial damage.

Emerging evidence indicates that even in non-plaque-induced gingival disorders such as DG, subtle microbial alterations may have significant immunological consequences. Epithelial disruption in these disorders increases antigenic exposure to subgingival microbiota, facilitating interactions between microbial ligands and pattern recognition receptors on epithelial and immune cells [2,3,7,55,56]. This activation not only enhances local immune surveillance but may also skew responses toward a pro-inflammatory phenotype, amplifying tissue destruction and perpetuating chronic disease. In this way, the microbiome may act as a silent amplifier of preexisting immune dysregulation, transforming a purely immunological disease process into a complex host–microbe interplay.

Taken together, these findings support the concept that the gingival microbiome is not merely a passive bystander but rather an active modulator of host immune responses in autoimmune-mediated mucosal diseases presenting with DG. By contributing to barrier dysfunction, antigen exposure, and immune activation, microbial communities may influence both the onset and the persistence of these conditions. Recognizing the microbiome as an integral component of DG pathogenesis opens new avenues for research and may ultimately inform diagnostic, prognostic, and therapeutic strategies that integrate microbial and immunological perspectives.

## 4. Microbiota in Immune-Mediated Mucosal Diseases

DG most frequently arises as a manifestation of immune-mediated mucocutaneous diseases such as OLP, MMP, and PV. These disorders have long been viewed as paradigmatic examples of immune dysregulation, involving autoreactive T cells, pathogenic autoantibodies, and chronic tissue inflammation. However, growing evidence indicates that oral microbial dysbiosis may contribute to their pathogenesis and persistence, either by amplifying immune activation or by interfering with mucosal barrier homeostasis. The microbiome is therefore increasingly considered not only as a secondary factor but as a potential co-driver of disease. Table 1 summarizes current evidence on microbial alterations, immune associations, and mechanistic hypotheses across OLP, MMP, and PV.

Among these conditions, OLP is by far the best studied in relation to microbial changes. Multiple sequencing-based investigations have demonstrated significant shifts in salivary and mucosal microbial communities of OLP patients compared with healthy controls. These studies consistently report enrichment of pro-inflammatory genera such as *Fusobacterium*, *Prevotella*, *Capnocytophaga*, and *Leptotrichia* [24,25,26,27,28,29,57,58,59,60]. In erosive and ulcerative forms, a marked reduction in microbial diversity has also been observed, suggesting that immune-mediated epithelial damage creates ecological niches favorable for opportunistic anaerobes. These dysbiotic communities promote inflammation through virulence factors, proteolytic activity, and metabolic products that compromise epithelial integrity and facilitate persistent biofilm formation. Importantly, such microbial changes are tightly linked with local immune alterations, including recruitment of neutrophils and T cells as well as activation of epithelial stress pathways [24,29,59,61,62].

At the immunological level, OLP-associated dysbiosis has been correlated with increased expression of pro-inflammatory cytokines such as IL-6, IL-17, and IL-23, together with enhanced recruitment of Th1 and Th17 subsets. Concurrently, activation of pattern recognition receptors such as TLR2 and TLR4 has been documented, highlighting the capacity of microbial ligands to sustain chronic inflammation [63,64,65]. This constellation of findings points to a self-reinforcing feedback loop in which dysbiotic microbiota stimulate epithelial and immune responses, while ongoing inflammation further destabilizes the microbial community. The result is a persistent cycle of mucosal damage and impaired resolution of inflammation that characterizes chronic OLP.

In contrast, direct microbiome studies in MMP and PV remain scarce, though several biologically plausible mechanisms have been suggested. In both diseases, epithelial barrier compromise creates direct access for microbes and their products to penetrate deeper tissues, thereby intensifying immune exposure to autoantigens. Secondary microbial colonization of erosive and ulcerative lesions may further sustain local inflammation, delay re-epithelialization, and promote antigenic mimicry between microbial and host structures [30,66]. Moreover, chronic pain, mucosal fragility, and patient reluctance to perform oral hygiene can exacerbate plaque accumulation, favoring shifts in the microbial ecosystem toward opportunistic pathogens [12,66,67].

In MMP, histological studies demonstrate early neutrophilic infiltration and heightened expression of inflammatory mediators even before frank blistering develops, raising the possibility that microbial stimuli could serve as early triggers or amplifiers of subepithelial immune activation [35,66]. In PV, although the autoimmune response is primarily mediated by antibodies against desmogleins, microbial antigens or microbial-induced cytokines such as TNF-α and IFN-γ may potentiate keratinocyte apoptosis and enhance acantholysis, thereby compounding tissue destruction [30,66,68,69]. These models underscore the potential for microbiome-derived signals to modulate disease course, even in conditions traditionally attributed to autoantibody-driven mechanisms.

Beyond taxonomic shifts, increasing attention has been directed toward the role of microbial metabolites. Biofilm-derived molecules, including short-chain fatty acids, hydrogen sulfide, and lipopolysaccharides, can profoundly influence mucosal immunity by modulating dendritic cell activation, shaping regulatory T-cell differentiation, and altering epithelial gene expression [70,71,72,73]. Such effects highlight a key mechanistic pathway through which microbial dysbiosis may undermine immune tolerance and promote sustained autoimmunity. Although direct mechanistic data in DG remain limited, the convergence of microbiological, immunological, and clinical findings strongly suggests that the oral microbiome is not merely a passive bystander but an active participant in the initiation, progression, and chronicity of immune-mediated diseases underlying DG.

## 5. Immune–Microbial Interactions in DG Pathogenesis

DG, particularly when arising in the context of OLP, MMP, and PV, exemplifies a multifactorial disease process in which host immune dysregulation and oral microbial shifts converge to perpetuate chronic mucosal injury. Rather than being passive reflections of underlying immune pathology, microbial communities may act as active co-modulators, shaping disease onset, severity, and persistence through continuous feedback interactions with epithelial and immune cells [2,10,11,12].

### 5.1. Dysbiosis and Epithelial Barrier Breakdown

**Figure 2:**
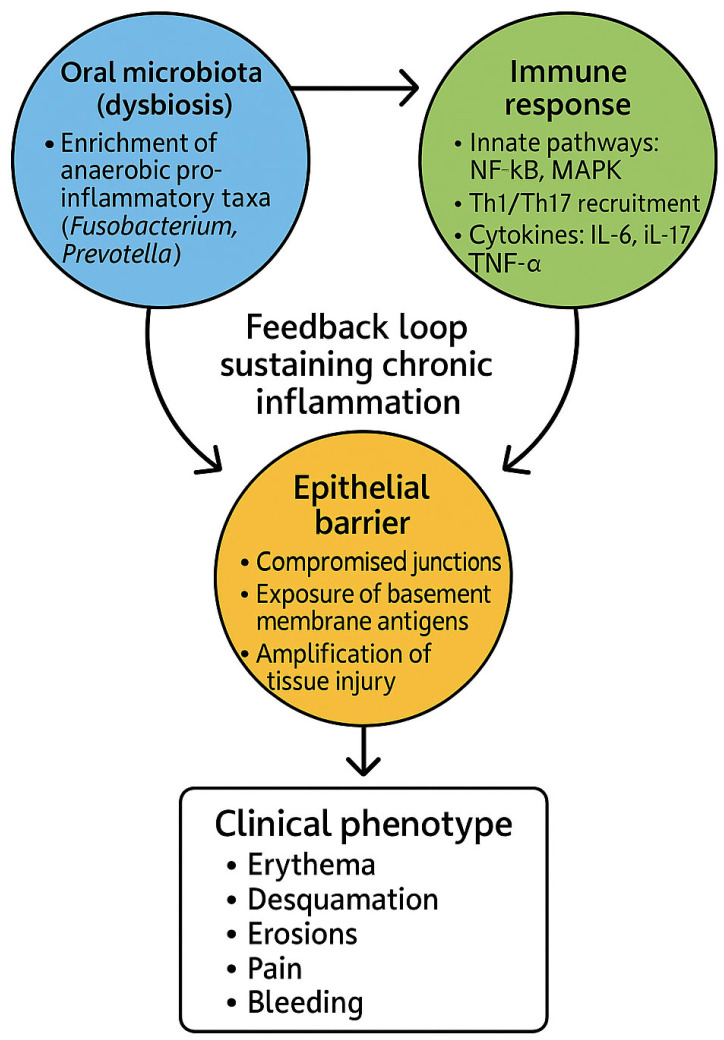
Conceptual model of microbiota–immune–epithelial interactions in desquamative gingivitis (DG). This schematic illustrates the dynamic interplay between dysbiotic oral microbiota, epithelial barrier integrity, and host immune responses in DG. Enrichment of anaerobic and pro-inflammatory taxa (e.g., *Fusobacterium*, *Prevotella*), together with the loss of protective commensals, compromises epithelial junctions and exposes basement membrane antigens. In parallel, microbial metabolites and pathogen-associated molecular patterns (PAMPs) activate innate immune pathways (e.g., NF-κB, MAPK) and promote recruitment of Th1 and Th17 cells, resulting in cytokine release (IL-6, IL-17, TNF-α) and chronic inflammation. These immune alterations amplify autoantibody-mediated tissue injury in conditions such as oral lichen planus (OLP), mucous membrane pemphigoid (MMP), and pemphigus vulgaris (PV). The resulting immune–microbiota feedback loop sustains epithelial damage, giving rise to the clinical phenotype of DG, characterized by erythema, desquamation, erosions, pain, and bleeding.

One of the earliest pathological events in DG is the loss of epithelial barrier integrity. In OLP, cytotoxic CD8^+^ T cells induce keratinocyte apoptosis at the epithelial–connective tissue interface, while in MMP and PV, autoantibody binding against basement membrane components or desmogleins produces subepithelial or intraepithelial blistering [1,2,3,5,74,75,76]. These processes expose previously sequestered antigens and permit direct microbial contact with subepithelial tissues. Dysbiotic biofilms enriched in Fusobacterium, Prevotella, and Capnocytophaga secrete proteases, hydrogen sulfide, and other virulence factors that degrade junctional complexes and extracellular matrices, further weakening the epithelial barrier [77,78,79]. The consequence is a vicious cycle in which barrier compromise facilitates microbial ingress, while microbial activity amplifies tissue injury and inflammatory signaling (Figure 2).

### 5.2. Activation of Pattern Recognition Receptors

Once the barrier is breached, microbial products such as lipopolysaccharides (LPS), fimbriae, and peptidoglycans directly engage PRRs on epithelial and immune cells. Among these, TLR2 and TLR4 play central roles in recognizing bacterial ligands and initiating downstream inflammatory cascades [80,81,82]. Activation of these pathways induces nuclear factor-κB (NF-κB) and mitogen-activated protein kinase (MAPK) signaling, leading to the production of IL-6, TNF-α, IL-17, and IL-23. These cytokines, which are consistently elevated in OLP and related DG-associated conditions, recruit Th1 and Th17 subsets, neutrophils, and cytotoxic CD8^+^ T cells to lesional sites [65,83,84,85]. The accumulation of these effector cells establishes a cytokine-rich milieu that sustains epithelial destruction, impairs mucosal healing, and drives lesion chronicity.

### 5.3. Loss of Tolerance and Autoimmunity

Chronic exposure to microbial and self-antigens within this inflammatory microenvironment can erode mechanisms of peripheral tolerance, particularly in genetically predisposed individuals. Molecular mimicry, whereby microbial antigens resemble host epitopes, may stimulate autoreactive lymphocytes, while persistent antigenic stimulation and bystander activation further expand autoimmune responses [86,87,88]. These processes may directly contribute to the generation of autoantibodies in PV and MMP or perpetuate aberrant T cell activity in OLP. Moreover, microbial metabolites such as short-chain fatty acids, indoles, and LPS can profoundly influence dendritic cell maturation and T-cell differentiation, shifting the balance from tolerogenic Tregs toward pro-inflammatory Th1 and Th17 subsets [89,90,91,92]. This skewed immune landscape explains why DG lesions are often refractory to therapy and prone to relapses, particularly in erosive OLP, where chronic antigen exposure and dysbiosis are most pronounced.

### 5.4. Inflammation–Dysbiosis Feedback Loops

The pathogenesis of DG can thus be conceptualized as a self-reinforcing loop. Inflammation alters the ecological conditions of the gingival niche, favoring microbes adapted to thrive in cytokine-rich, oxygen-depleted, and protease-abundant environments. These dysbiotic communities, in turn, release factors that intensify epithelial barrier breakdown and immune activation, thereby sustaining the inflammatory cycle (Figure 2). This bidirectional model parallels mechanisms described in inflammatory bowel disease and psoriasis, highlighting a broader paradigm of mucosal immunopathology in which dysbiosis and immune dysfunction are inseparably intertwined [53,54,93,94]. Importantly, this perspective underscores that DG should not be viewed solely as an immune-mediated disease, but rather as the product of a dynamic host–microbe ecosystem where both partners contribute to disease chronicity.

## 6. Clinical and Therapeutic Implications

Understanding the complex interplay between the oral microbiome and immune-mediated processes in DG creates new opportunities for precision diagnostics, monitoring, and therapy. Since DG-associated diseases are often chronic, relapsing, and partially resistant to conventional immunosuppressive treatment, incorporating microbial considerations into clinical decision-making may improve both disease control and patient quality of life. This integrative approach not only emphasizes suppression of aberrant immunity but also the stabilization of the microbial ecosystem that continually interacts with the gingival mucosa.

### 6.1. Impact of Immunosuppressive Therapy on Microbial Ecology

Conventional therapy for DG relies primarily on topical or systemic corticosteroids, with escalation to immunosuppressants such as azathioprine or mycophenolate mofetil, and biologic agents like rituximab in severe or refractory cases [3,95,96,97]. These interventions are highly effective in dampening T-cell activation, autoantibody production, and downstream inflammation, thereby providing symptomatic relief and mucosal healing. However, their impact extends beyond immune suppression. Corticosteroids, for example, reduce salivary antimicrobial peptides and impair epithelial turnover, weakening mucosal defenses against opportunistic microbes [98,99,100]. This ecological shift predisposes patients to *Candida* overgrowth and secondary infections, which not only worsen discomfort but may also obscure clinical assessment of disease activity [101,102]. Similarly, broad immunosuppressants and B-cell–depleting agents can disrupt immune surveillance, further destabilizing oral microbial homeostasis and prolonging epithelial barrier dysfunction [101,102,103]. Thus, while immunosuppressive therapies are indispensable in controlling inflammation, their collateral effects on microbial ecology highlight the need for careful monitoring and, potentially, adjunctive antimicrobial or microbiome-stabilizing strategies.

### 6.2. Microbiome-Modulating Therapies: A Future Avenue?

Given the recognized role of dysbiosis in DG pathogenesis, microbiome-modulating interventions are gaining traction as possible adjuncts to conventional immunosuppression. Topical antiseptics, including chlorhexidine and cetylpyridinium chloride, are commonly employed to control biofilm burden and reduce gingival inflammation [104,105,106]. However, recent evidence indicates that prolonged use of chlorhexidine can disrupt commensal microbial communities and promote oral dysbiosis, highlighting the need for cautious and short-term application [107]. In addition, essential oils such as thymol, eucalyptol, and menthol exhibit broad-spectrum antibacterial and antifungal properties and may support microbial modulation when incorporated into mouthrinses or topical formulations, although clinical data in DG remain scarce [108,109]. Yet, prolonged use has been associated with ecological disruption of commensal species, mucosal irritation, and even emergence of antiseptic-resistant phenotypes [110,111]. To overcome these limitations, more selective approaches have been proposed. Narrow-spectrum antimicrobials and bacteriophage therapy could theoretically target pathogenic organisms such as *Fusobacterium* or *Prevotella* while sparing beneficial taxa, though such interventions remain largely experimental [12,51,112].

Another promising strategy is the use of probiotics. Certain *Lactobacillus* (e.g., *L. rhamnosus*, *L. reuteri*, *L. salivarius*) and *Streptococcus* strains (e.g., *S. salivarius* K12, *S. oralis*) have shown potential to restore microbial balance, downregulate pro-inflammatory cytokines, and promote epithelial regeneration in oral disease models [12,113]. Beyond live organisms, the therapeutic exploitation of microbiome-derived metabolites represents an emerging frontier. Short-chain fatty acids, particularly butyrate and propionate, exert immunoregulatory effects by enhancing Treg activity and suppressing Th17-driven inflammation, while tryptophan catabolites such as indole-3-propionic acid have been implicated in strengthening epithelial barrier function [114,115,116]. Harnessing these molecules, either through dietary modulation, metabolite supplementation, or engineered probiotics, may provide innovative adjunctive options for managing refractory or recurrent DG.

### 6.3. Microbiome Profiling in Diagnosis and Monitoring

The advent of next-generation sequencing and high-throughput microbial profiling has transformed the capacity to investigate oral ecosystems in health and disease. Applied to DG, salivary or plaque microbiome analysis could enable differentiation between disease subtypes such as erosive OLP and MMP, where distinct dysbiotic patterns have been described [3,59,79]. Early detection of pro-inflammatory shifts or loss of microbial diversity may also help identify patients at risk of more aggressive disease progression [43]. Beyond cross-sectional assessment, longitudinal monitoring of the microbiome during therapy could provide dynamic biomarkers of response, with restoration of diversity and reduction in pathogenic taxa serving as surrogate indicators of remission or treatment success [117].

Importantly, microbiome-based diagnostics are unlikely to replace conventional histopathology and immunofluorescence, but rather complement them. By integrating microbial signatures with clinical and immunological parameters, a precision medicine framework can be developed in which patient-specific microbial data inform tailored treatment strategies. Such an approach holds promise not only for improving disease control in DG but also for minimizing unnecessary immunosuppression, reducing treatment-related complications, and optimizing long-term patient outcomes.

## 7. Research Gaps and Future Directions

**Figure 3:**
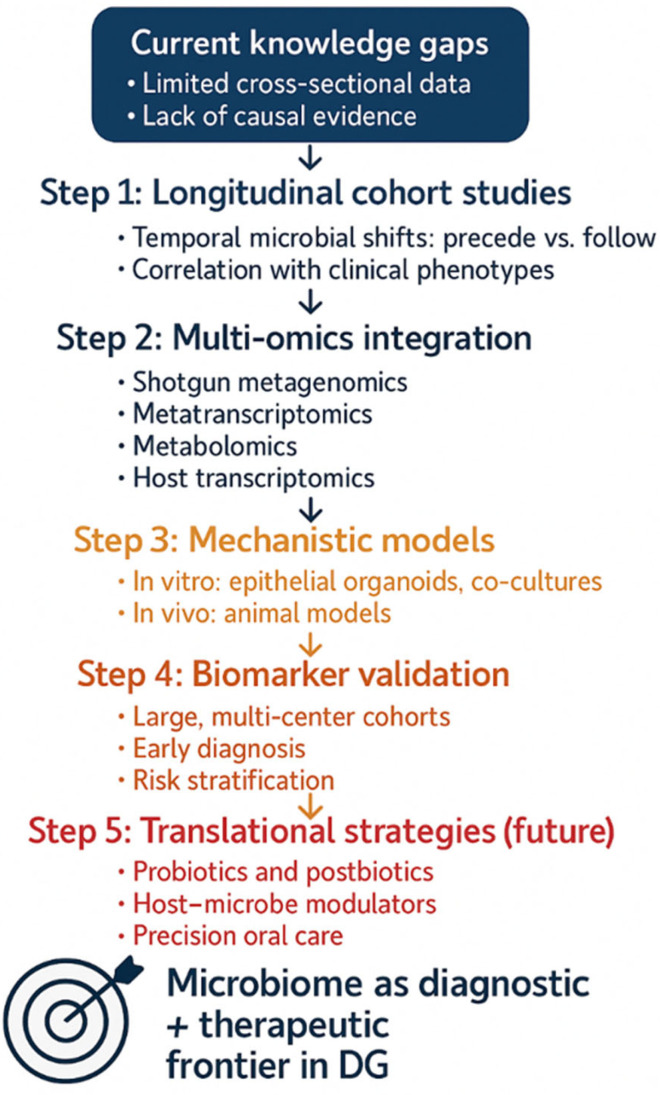
Research roadmap for microbiome studies in desquamative gingivitis.

Despite increasing recognition of the oral microbiome’s role in immune-mediated mucosal diseases, DG remains critically underexplored from microbiological, immunological, and systems biology perspectives. Current evidence provides only a fragmented picture, with significant limitations in scope and methodology. Addressing these shortcomings is essential to move beyond descriptive associations toward actionable insights that can inform diagnosis, monitoring, and therapy (Figure 3).

First, comparative microbiome studies across DG subtypes are virtually nonexistent. Most published investigations have centered on OLP, reflecting its higher prevalence and established association with DG [2,3]. By contrast, MMP, PV, and other rarer conditions remain poorly characterized in terms of microbial alterations, despite sharing similar clinical manifestations. Systematic reviews have repeatedly highlighted this imbalance, emphasizing the need for cross-disease studies that could disentangle common microbial denominators from condition-specific dysbiotic signatures [30,66]. Such comparative datasets would be invaluable not only for understanding disease mechanisms but also for developing microbiome-based diagnostic tools that can refine differential diagnosis and risk stratification in clinically overlapping presentations.

Second, the majority of available studies are cross-sectional, limiting their ability to infer temporal relationships between dysbiosis and disease activity. Although mechanistic data support a self-reinforcing inflammation–dysbiosis feedback loop, the chronological sequence of these events in DG remains unclear. It is therefore unresolved whether microbial alterations primarily act as triggers that initiate inflammation, amplifiers that sustain it, or consequences of tissue damage. Longitudinal studies incorporating sampling during flares, remission, and post-treatment are essential to determine whether microbial shifts precede clinical exacerbations, mirror therapeutic response, or represent secondary colonization of already compromised epithelium [117,118]. This temporal uncertainty does not contradict the feedback model but rather emphasizes the need to define its onset and directionality in vivo.

Third, functional characterization of the microbiome in DG is rudimentary. Most existing data derive from 16S rRNA profiling, which provides taxonomic information but limited insight into microbial activity. Functional omics approaches—including shotgun metagenomics, metatranscriptomics, and metabolomics—are urgently needed to identify virulence genes, metabolic pathways, and biofilm-related factors relevant to DG [70,71,72,73,119,120,121,122]. For example, characterization of metabolites such as short-chain fatty acids or hydrogen sulfide could illuminate mechanisms of immune modulation and epithelial barrier disruption, while transcriptomic analysis may uncover actively expressed microbial pathways during disease flares. Integrating these layers would advance the field from cataloguing “who is there” to deciphering “what they are doing.”

Fourth, the adoption of systems immunology frameworks is necessary to fully elucidate host–microbe interactions in DG. Single-modality studies risk oversimplifying what is inherently a networked process involving microbial signals, epithelial integrity, and immune pathways. Future research should therefore combine microbial metagenomics with host transcriptomics, flow cytometric immune phenotyping (e.g., Th1/Th17/Treg balance, TLR expression), and functional barrier assays [123,124,125]. Such multi-omic triangulation could pinpoint causal mechanisms, identify robust biomarkers, and generate predictive models of disease course, moving the field closer to precision medicine.

Fifth, microbiome-targeted interventional studies are virtually absent in DG. While theoretical frameworks support probiotics, prebiotics, bacteriophages, or small-molecule modulators as adjuncts to immunosuppression, there is little empirical evidence regarding their efficacy or safety in this patient population [51,126]. Pilot clinical trials are needed to test feasibility, optimize delivery methods, and establish whether such interventions can reduce relapse rates, shorten healing times, or enhance quality of life in refractory DG. Importantly, these studies should include immunological and microbial endpoints to clarify mechanisms of action.

Finally, methodological rigor and standardization must be prioritized. Current heterogeneity in sample collection (saliva, plaque, gingival swabs, or biopsy-associated samples), storage conditions, DNA extraction protocols, and sequencing platforms severely limits comparability across studies [127,128]. Similarly, clinical metadata are often underreported, including disease subtype, treatment status, or confounders such as antibiotic use, smoking, or diet—all of which significantly shape microbial composition. Establishing standardized protocols and transparent reporting frameworks will be critical for building reproducible, interoperable datasets that can drive meta-analyses and translational applications.

In summary, addressing these gaps through well-designed, multidisciplinary studies will be pivotal for advancing our understanding of immune–microbial crosstalk in DG. Such efforts hold the promise of moving from descriptive associations toward microbiome-informed diagnostics and therapeutics, ultimately transforming the management of DG from empiric immunosuppression to targeted, precision-based interventions.

## 8. Conclusions

DG represents a clinically challenging and immunologically complex manifestation of several mucocutaneous disorders, most prominently OLP, MMP, and PV. Traditionally interpreted as the end-product of immune dysregulation, it is now increasingly evident that the oral microbiota may contribute to disease onset, chronicity, and severity through dynamic interactions with the epithelial barrier and the host immune system.

In OLP, the best-studied DG-associated condition, consistent features of dysbiosis have been observed, characterized by enrichment of pro-inflammatory genera such as *Fusobacterium* and *Prevotella* alongside reduced microbial diversity. These microbial alterations appear to mirror clinical severity, especially in erosive forms, and are mechanistically linked with heightened expression of inflammatory cytokines and recruitment of pathogenic T-cell subsets. By contrast, data on MMP, PV, and other conditions remain limited. Nonetheless, converging pathophysiological themes—including epithelial barrier fragility, chronic mucosal inflammation, and secondary microbial colonization of erosive surfaces—provide biological plausibility for a microbiota-driven modulatory role across these entities.

Mechanistically, DG may represent a prototypical example of host–microbiome–immune crosstalk gone awry. Barrier compromise facilitates deeper microbial penetration, microbial ligands activate pattern recognition receptors, and immune dysregulation drives cytokine imbalances and loss of peripheral tolerance. Together, these processes form self-amplifying feedback loops that perpetuate epithelial injury and sustain chronic inflammation. Such a model aligns DG with broader paradigms of mucosal immunopathology observed in gastrointestinal and dermatological autoimmune diseases, underscoring the systemic relevance of oral microbiome research.

From a clinical standpoint, these insights offer a roadmap for future innovation. Microbiome-informed diagnostics may enable refined disease subtyping, risk stratification, and real-time monitoring of treatment response. In parallel, therapeutic strategies aimed at microbiome modulation—whether through targeted antimicrobials, probiotics, bacteriophage therapy, or metabolite-based interventions—hold promise as adjuncts to conventional immunosuppressive regimens. Importantly, these approaches may not only enhance therapeutic efficacy but also mitigate treatment-related complications such as secondary infections, thus improving overall quality of life for affected patients.

Yet, the field remains in its infancy. Key uncertainties persist regarding causality, the specificity and stability of microbial signatures, and the translational feasibility of microbiome-based interventions. Addressing these challenges will require longitudinal studies that track microbial and immune dynamics over time, functional and multi-omics approaches to identify mechanistic pathways, and rigorously designed interventional trials to test microbiome-targeted therapies in DG.

Ultimately, integrating microbiome science into the diagnostic and therapeutic framework of DG offers the prospect of more precise, personalized, and durable care. However, it must also be acknowledged that current evidence is primarily associative, and the oral microbiome may represent a secondary bystander rather than a primary driver of disease in some cases. Future studies should therefore rigorously test both causal and null hypotheses to delineate whether microbial alterations are initiating, modulating, or merely reflecting ongoing immune pathology. By moving beyond the current paradigm of empiric immunosuppression toward a model that accounts for both immune and microbial determinants, while remaining open to alternative explanations, future research may more accurately define the role of the microbiome in DG and related immune-mediated mucosal disorders.

## Figures and Tables

**Table 1:** Summary of oral microbiome findings in immune-mediated diseases associated with desquamative gingivitis.

Disease	Microbiome Alterations	Immune Associations	Key References
Oral lichen planus (OLP)	↑ Pro-inflammatory genera (*Fusobacterium, Prevotella, Capnocytophaga, Leptotrichia*); ↓ microbial diversity (erosive/ulcerative OLP); dysbiotic enrichment in anaerobic & proteolytic taxa	↑ IL-6, IL-17, IL-23; Th1/Th17 recruitment; TLR2/TLR4 activation; microbe–immune feedback loop sustaining mucosal inflammation	[24,25,26,27,28,29,57,58,59,60,61,62,63,64,65]
Mucous membrane pemphigoid (MMP)	Very limited direct studies; potential secondary colonization of erosive lesions; impaired oral hygiene → plaque accumulation, opportunistic pathogens; however, available data are limited by small sample sizes and lack of standardized microbiome sequencing protocols	Early neutrophil infiltration; upregulation of inflammatory mediators; microbial stimuli may trigger/amplify subepithelial inflammation	[12,30,35,66,67]
Pemphigus vulgaris (PV)	Sparse microbiome data; epithelial barrier breakdown enables microbial infiltration; secondary dysbiosis of erosions likely; current evidence remains limited by the absence of longitudinal or comparative studies exploring microbial shifts during disease activity and remission	Autoantibody-driven acantholysis exacerbated by microbial antigens & cytokines (e.g., TNF-α, IFN-γ)	[30,66,68,69]

↑ meaning “elevated”; ↓ meaning “reduced”; → meaning “leads to”.

## Data Availability

Not applicable.

## Abbreviations

The following abbreviations are used in this manuscript:

CD8 + T cells	Cytotoxic T lymphocytes
DG	Desquamative gingivitis
DIF	Direct immunofluorescence
DNA	Deoxyribonucleic acid
IFN-γ	Interferon gamma
IL	Interleukin (IL-6, IL-17, IL-23 mentioned specifically)
MAPK	Mitogen-activated protein kinase
MMP	Mucous membrane pemphigoid
NF-κB	Nuclear factor kappa-light-chain-enhancer of activated B cells
OLP	Oral lichen planus
PAMPs	Pathogen-associated molecular patterns
PRRs	Pattern recognition receptors
PV	Pemphigus vulgaris
Th1	T helper 1 cells
Th17	T helper 17 cells
TLRs	Toll-like receptors
TNF-α	Tumor necrosis factor alpha
Tregs	Regulatory T cells
